# Primary Phenomenon in the Network Formation of Endothelial Cells: Effect of Charge

**DOI:** 10.3390/ijms161226149

**Published:** 2015-12-07

**Authors:** Shunto Arai

**Affiliations:** Department of Applied Physics, Graduate School of Engineering, The University of Tokyo, Hongo 7-3-1, Bunkyo-ku, Tokyo 113-8656, Japan; arai@ap.t.u-tokyo.ac.jp; Tel./Fax: +81-3-5841-7757

**Keywords:** endothelial cells, network formation, simulation method, charged particle system, fluid dynamics

## Abstract

Blood vessels are essential organs that are involved in the supply of nutrients and oxygen and play an important role in regulating the body’s internal environment, including pH, body temperature, and water homeostasis. Many studies have examined the formation of networks of endothelial cells. The results of these studies have revealed that vascular endothelial growth factor (VEGF) affects the interactions of these cells and modulates the network structure. Though almost all previous simulation studies have assumed that the chemoattractant VEGF is present before network formation, vascular endothelial cells secrete VEGF only after the cells bind to the substrate. This suggests VEGF is not essential for vasculogenesis especially at the early stage. Using a simple experiment, we find chain-like structures which last quite longer than it is expected, unless the energetically stable cluster should be compact. Using a purely physical model and simulation, we find that the hydrodynamic interaction retard the compaction of clusters and that the chains are stabilized through the effects of charge. The charge at the surface of the cells affect the interparticle potential, and the resulting repulsive forces prevent the chains from folding. The ions surrounding the cells may also be involved in this process.

## 1. Introduction

Blood vessels are ubiquitous in vertebrates and are essential organs responsible for the supply of oxygen and nutrients to the whole body. Elucidation of the mechanism of vasculogenesis and angiogenesis is crucial for our understanding of oxygen transport, organ development, and wound healing. Although many studies have examined the formation of vascular networks [1,2,3,4], our understanding of these networks is still far from complete.

Previous studies have proposed various simulation methods based on the authors’ assumptions of properties of network formation, such as chemotaxis [5,6], elongated cell shapes [7,8,9], optimization for flows in blood vessels [10], and interactions among cells and related components [11,12]. Almost all of these simulation studies consider final equilibrium configurations and state the importance of the concentration gradient of vascular endothelial growth factor (VEGF). VEGF is a protein produced by cells after they adhered to the substrate; however, simulation studies usually assume the presence of VEGF from the initial random state [5,6]. In addition, the experimental measurement of the diffusion coefficient of VEGF [13] suggests that the diffusion coefficient obtained from the experiments is quite large compared to the value which is assumed in some simulation studies. This indicates the gradient of VEGF concentration is not essential for the vascular network formation especially at the early stage. Thus, the initial configurations of such simulation models have not been calculated properly, and the final configurations must therefore also be incorrect. If there exist some precursors of network, this intermediate stable state affects the kinetic selection for the final stable configuration. In order to identify the kinetic pathway for network formations, it is necessary to perform dynamic simulations which treat the equations of motion and fluctuation properly. In addition, the concentration gradient of VEGF may have minimal effects during the initial stage of network formation owing to temperature inhomogeneity and the vibration of the microscope stage [8]. Therefore, we focused on the initial stage of network formation by vascular endothelial cells, during which precursors of networks are formed through purely physical means, such as the flow of solvents, the Brownian motion of cells and ions surrounding cells, and the electrostatic interactions among cells. In particular, the repulsive force conferred by the surface charge of the cell membrane may mainly affect the configuration of the network; however, these factors have generally been ignored in previously reported simulations, and the effects of ions and the flow of the solvents surrounding the cells have not been properly modeled owing to its difficulty in treating these factors.

In order to consider primary cluster formation of vascular endothelial cells and to clarify the effect of VEGF, we performed *in vitro* experiment which was absence of VEGF as a model system. Although *in vitro* experiments do not permit studies of actual interactions and morphogenesis *in vivo*, they are quantifiable and reproducible, and they often provide meaningful data, which helps to understand *in vivo* network formation [6,11]. From these viewpoints, we conducted an experiment *in vitro* with the culture condition absence of VEGF. Based on the experimental results we also introduce herein a purely physical model and a simulation method for charged-particle systems. This allowed us to consider not only the dynamics of particles but also dynamic coupling with solvents and ionic clouds surrounding the cells.

### Network Formations of Endothelial Cells

**Figure 1:**
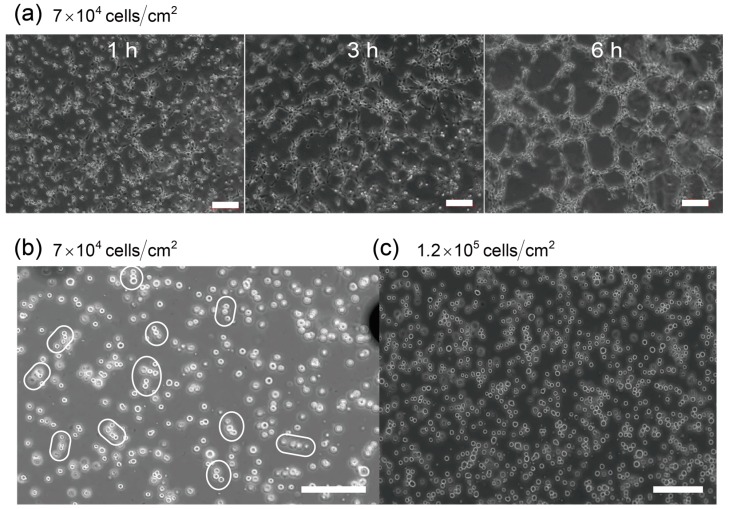
(**a**) Network formation of vascular endothelial cells at 6 h after dispersion of cells. When the areal fraction of cells was sufficiently high to distribute throughout the system (above the percolation threshold [8]), the cells first shrunk and then formed networks; (**b**) As a primary phenomenon of network formation, chain-type clusters were formed before cells adhered to the substrate. When the number of these increased, the chains formed percolated networks, as shown in (**a**); (**c**) In the case of increased cell density, cells still tended to form chains, and the coarsening seems to be stopping for a while. These photos are taken by Matsunaga [14]. Both of the photos, (**b**,**c**), are taken at 10 min after cells were dispersed into the medium. All of the experiments above were conducted with the culture in the absence of VEGF (vascular endothelial growth factor). All scale bars indicate 200 μm.

Many previous studies of network formation of cells have focused on the later stages (≥1 h) in order to observe the dynamics of network formation of cells accompanied by dramatic structural changes (Figure 1a). Almost all of these studies have assumed that VEGF, which functions as a chemoattractant and a signal protein which stimulates network formations, is present. However, cells cannot secrete VEGF before adhering to the substrate. In this paper, we propose a purely physical model for cluster formation of vascular endothelial cells with a focus on the early stages of network formation (≤20 min). In order to identify the precursors of these cell networks, we prepared cells and substrates and made simple observations of the suspensions using a phase-contrast microscope. The following experiments were conducted with a culture that did not contain VEGF in order to identify the role of VEGF. The brief method is shown in Supplemental Information, and the details are shown elsewhere [14].

From the observations we could track the cells during the early stages (after 10 min) of network formation (Figure 1b). During this stage, the cells formed clusters with chain-like shapes (indicated by the white circles in Figure 1b). These clusters were not affected by the concentration gradient of chemoattractants, such as VEGF. In the late stages of network formation, these chains shrunk, and cells began to form networks. Therefore, the chain appeared to act as precursors for the networks. Note that this chain formation is not limited to our experimental results, since these chains can also be seen in the results of Serini [6] and Tosin [11]. Thus we believe these chain formation is observed universally in *in vitro* experiments. In addition, even if the number of cells was increased, the cells did not aggregate or form compact spherical clusters. Notably, the cells did still form chains, although the compact spherical clusters should be the energetically stable state if the interaction among cells is only the attractive forces, indicating that the formation of chains was strongly affected by kinetic processes. In the next section, we show the results of simulation whose details are introduced in Section 3, and we also discuss the mechanisms of this chain formation by applying the simulation method in Section 2.

## 2. Results and Discussion

Previous simulations of network formation by endothelial cells can be classified into three types [3,4,15]:

(1) Cellular Potts model
(2) Continuous model
(3) Lattice free particle dynamics

The first model (cellular Potts model) is a lattice-based computational method and is widely used to describe the network formation of endothelial cells [16]. In this simulation, each point moves according to a set of rules. This method allows us to change the total volume of cells and consider many types of interactions acting on each lattice. However the motility of cells cannot be addressed properly because cells are treated as continuous coarse-grained field, and the time-evolution of the cells are stochastically decided and thus the motility of cells becomes the same as the surrounding chemicals. The same features are observed in the continuous model. In this model, we can calculate the dynamical interactions and the distribution of chemicals such as chemotaxis [17], hydrodynamic interactions [18] and viscoelastic effects [19] which is introduced by using the stress-diffusion coupling scheme [20]; however, the cells are also treated as a concentration field, and thus, the motility of the cells is fast, regardless of the differences in size between cells and fluids (or chemicals).

These difficulties come from the assumption that cells are not treated as particles with macroscopic sizes but as a continuous density field. Based on this observation, the third method, lattice-free particle dynamics, appears to be suitable for simulation of the dynamic behavior of network formation, despite the difficulty of introducing other degrees of freedom, such as fluids and chemicals surrounding the cells [8,21]. Thus, we chose a hybrid method, combining the continuous model and the lattice-free model, in order to properly model the dynamics of the system. This method is called as fluid particle dynamics (FPD) method [22]. The detail is shown in Section 3.1.

### 2.1. Results of FPD Simulation Including the Effect of Surface Charge of Cells

**Figure 2:**
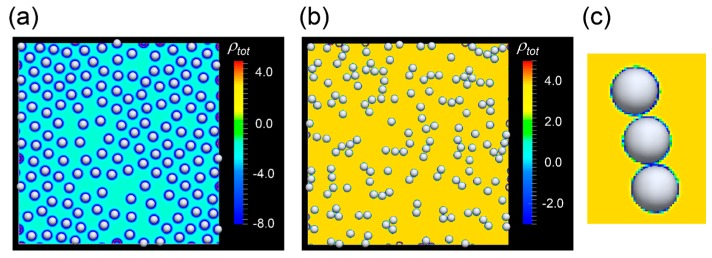
Morphogenesis of vascular endothelial cells. White spheres indicate cells, and the color shows the sum of concentration of ions ρ_tot_: (**a**) without salt (only the counter ions of cells are considered) and (**b**) with salt (Cα = 20 μM). This corresponds to the early stage of vascular network formation; (**c**) Expanded View of a chain composed by three cells. Cells are surrounded by ions denoted by the color field which is the same as (**b**).

The results of many body two-dimensional (2D) simulations are shown in Figure 2. In these simulations, we set the area fraction to $\phi_{\text{area}}=0.15$. When the system does not contain salts for screening the interaction, cells are dispersing by the long-range Coulomb repulsion, and if the electrostatic interactions are sufficiently large, cells form a Wigner crystal like structure (Figure 2a). On the other hand, when we add salts to screen the interaction, the spherical cells aggregate and form clusters of chain-like shapes (Figure 2b), as was observed in the experimental results of endothelial cells. The experimental results in Figure 1b,c show that the chain-like clusters consist of 3–5 cells, which is appropriately described in the Figure 2b. From these results, we confirm that the electrostatic force is controlled by the ions and that the range of interaction becomes shorter as the concentration of ions increased. This in turn accelerates the formation of chains of cells.

### 2.2. Effects of Hydrodynamic Interactions on the Formation of the Chain-Like Structure

In a system of charged cells dispersed into the medium, linear chain structures were selected as the stable structure, though compact clusters became the more energetically stable structures, minimizing the attractive potential acting on cells. Without chemotaxis, there were two main reasons for the selection of this dynamic structure: (1) the hydrodynamic effect; and (2) the effect of charge.

The hydrodynamic interaction can alter the kinetic pathway, as in phase separation of binary mixtures. The phase separations of binary solid and liquid mixtures are described by the framework of the universality class as models B and H, respectively [23]. In the model B simulation, spherical droplets emerge from the initial stage of phase separation as stable structures under asymmetrical compositions. In the model H simulation in which hydrodynamic interactions are considered, continuous drops with an elongated shape persist longer. This difference suggests the hydrodynamic interactions play a role in the chain (or elongated shape) formation of cells. In order to clarify the hydrodynamic effects, we compared the aggregation structures using two distinct simulation methods: (1) Langevin dynamics (LD) simulation; and (2) FPD simulation. The former method ignores the effects of hydrodynamic interactions (see Section 3.4), whereas the latter considers the interaction as shown in Section 3.1. In this section, we focus on the effects of hydrodynamic interactions and ignore the effects of charge.

**Figure 3:**
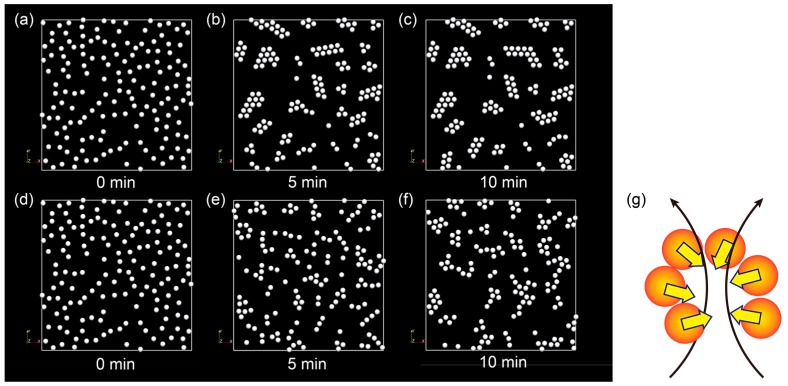
Effects of hydrodynamic interactions: (**a**–**c**) Langevin dynamics simulation (without hydrodynamic interactions); and (**d**–**f**) fluid particle dynamics simulation (including the effects of long-range hydrodynamic interactions); (**g**) Schematic showing the deceleration of chain folding. To form the spherical cluster, it is necessary to squeeze the fluid surrounding cells, which slows the dynamics of the interactions. This dynamic selection supports the chain-like nature of clusters, despite the observation that the most stable structure is the spherical cluster.

The results of comparisons between LD and FPD are shown in Figure 3. LD simulation reveals that compact clusters are formed during early stages of network formation as the energetically stable state (Figure 3a–c). In contrast, the FPD simulation yields long-lived linear clusters (Figure 3d,e). These results indicate that hydrodynamic interactions play an important role in decelerating the compaction of clusters. When cells approach each other, the solvent found in between the cells has to be squeezed out, as shown in Figure 3g. We speculate that this finite volume effect of the cells establishes the barrier for compaction and that the hydrodynamic interactions will slow the dynamics of aggregation, as in the coil-globule transition of a polymer or the formation of colloidal gels [24,25,26].

Although the coarsening process slows owing to the hydrodynamic interactions, the final configuration simulated by the FPD method, as shown in Figure 3f, becomes compact and is therefore still thicker than that observed in the experimental results in cells. This suggests that there may be another force suppressing the compaction of cells. We speculate that this other force may be a short-range repulsive force, such as Coulomb force. Indeed, the results shown in Figure 2b clearly suggests that the chain structure is achieved without the effects of chemotaxis. In the next section, we will consider the effects of charge on network formation in vascular endothelial cells.

### 2.3. Effects of Charge on the Formation of Chain-Like Structures

**Figure 4:**
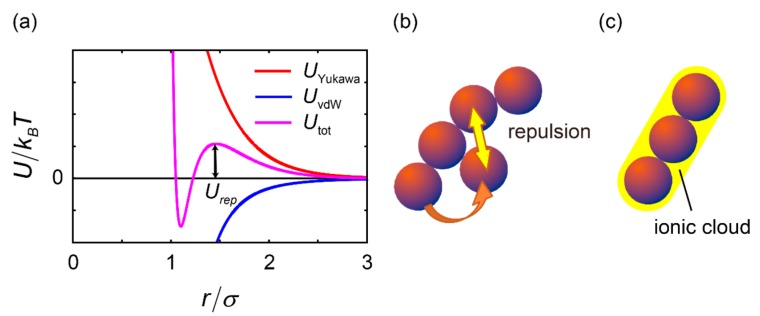
Chain-like cluster formation. (**a**) Schematic explanation of an effective potential acting on cells. The blue and red lines show the electrostatic repulsion and integrated van der Waals attractions, respectively; (**b**) The long-range nature of repulsive Coulomb interactions stabilized the chain by preventing the clusters from folding; (**c**) Ionic clouds surrounding cells may also play an important role in making the chain-like structure rigid.

In the previous section, we showed that hydrodynamic interactions increased the lifetime of the chains. We also showed the chain structures were stabilized by Coulomb repulsive forces, as shown in Figure 2b. This could be explained by the effects of an effective pair potential acting on the cells or the effects of ions surrounding the cells. The effective potential is shown in Figure 4a. Competition between the attractive and repulsive potentials is shown. The small repulsive force *U*_rep_ shown in Figure 4a prevented the chain from folding, as explained schematically in Figure 4b. In addition, the ionic cloud surrounding the cells supported the predicted rigidity of the chains (Figure 4c). Cells had a relatively high surface charge [27,28], and these charges bound the ions close to the cell surface. As shown in Figure 2c, ionic clouds (shown in blue) covered the chains, making the structures rigid by repelling other ions through weak interactions schematically described in Figure 4c (shown in yellow).

### 2.4. Other Systems for Chain Formation

**Figure 5:**
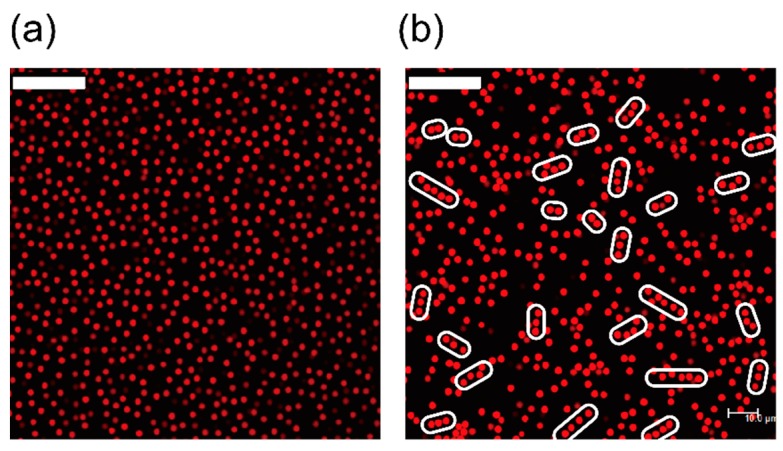
Observation of charged colloids. Charged colloids are used as a model system, and are observed using confocal microscope. (**a**) Colloids are dispersed in the medium without salt. Colloids do not form chain-like clusters, and this phase corresponds to Figure 2a; (**b**) Chain-like structure in a simple system. The colloidal dispersion, including salts, which screened the interactions of charged colloids (diameter: 2.6 μm), also exhibited the same configuration as cells, indicating that this structure formed without the assumptions of interactions and chemical reactants. Scale bars show 25 μm.

The results of simulation and our assumptions about the reasons for chain formation suggested that chains did not originate from chemotaxis but through a purely physical interaction. This was confirmed by a simpler system in which the particles interacted not only through an attractive force but also through a repulsive force. To test this, we performed experiments with monodispersed polymethyl methacrylate (PMMA) colloids (diameter: 2.6 μm) dispersed in bromocyclohexane (CHB, Sigma-Aldrich, St. Louis, MO, USA). The colloids were synthesized according to the method described by Antl and Bosma [29,30], and polydimethylsiloxane (PDMS, Gelest, Inc., Frankfurt am Main, Germany) was used as the steric stabilizer [31]. The refractive index of these colloids was close to that of CHB. This design allowed us to capture images clearly using a confocal laser-scanning microscope (Leica SP5, Leica Microsystems, Wetzlar, Germany). When the system includes no salts, the long-range Coulomb repulsion was stronger than the attractive force, and the colloids achieved stable dispersions (see Figure 5a), similar to that observed in Figure 2a. However, once the system included salts (e.g., tetrabutylammonium bromide (TBAB, Fluka, Sigma-Aldrich, St. Louis, MO, USA)), the Coulomb repulsion was screened, and the colloids formed chain-like clusters (Figure 5b). Owing to the similar refractive indices of solvents and particles, the van der Waals attraction between the colloids was quite small in these experiments according to the McLachlan equation [32]; however, the same trend was observed as the simulation results, and the colloids did not form compact clusters. In order to form the longer, more stable chains with colloids, highly charged colloids with large diameters or colloids having refractive indices different from those of the solvents should be used.

## 3. Materials and Methods

### 3.1. Simulation of Charged Particles

When solvents or other degrees of freedom are introduced, a solid-fluid boundary condition should inevitably be considered. In order to overcome this difficulty, we consider cells as highly viscous fluid drops having a nondeformable shape. Thus, in this model, cells are treated as immiscible drops, and we could properly model hydrodynamic interactions acting on cells. This also resulted in lower costs associated with simulation of the solid–liquid boundary condition. This method, called the fluid particle dynamics (FPD) method, was first introduced by Tanaka *et al.* [22], and a similar method was proposed by Yamamoto *et al*. [33]. For simplicity, we treated the cells as spheres and moving with Brownian motion, and their positions were developed under mesh-free conditions, as is applied in molecular dynamics (MD) simulations.

In order to distinguish the motility of cells and solvents, we introduce the viscosity field

(1) $$\eta \left(r\right)=\eta_{sol}+\sum_{i=1}^{PN}\left(\eta_{cell}-\eta_{sol}\right)\phi_{i}\left(r\right)$$

where η_*cell*_ is the viscosity inside a cell, and η_*sol*_ is the viscosity of the solvent surrounding the cells. To calculate this viscosity field, a particle is represented by the concentration field $\phi_{i}\left(r\right)$. According to the original FPD simulations, we set $\phi_{i}\left(r\right)=\left[\text{tanh}\left\{\left(a-\left|r-r_{i}\right|\right)/\xi \right\}+1\right]\;/\;2$, where *a* is the radius of a particle, and *ξ* is the interfacial thickness, set to *ξ* = 500 nm. For the limits of $\eta_{cell}/\eta_{sol}\to \infty$ and ξ/a→0, cells are regarded as a rigid solid; however, in our simulation, the unit of time is related to the viscosity of solvents, and we used η_*cell*_/η_*sol*_ = 50 and ξ/a=0.2 (in 3D), ξ/a=0.1 (in 2D) to reduce the computation time. These parameters are guaranteed to satisfy the requirement that the fluid particles show solid-like behavior [22].

The equation of motion is given by the Navier–Stokes equation:

(2) $$\rho \left(\frac{\partial }{\partial \;t}+v\cdot \nabla \right)v=f-\nabla p+\nabla \cdot \left[\eta \left(r\right)\;\left\{\nabla v+{\left(\nabla v\right)}^{t}\right\}\right]+f^{B}$$

where *ρ*, *v* and *p* denote the density, velocity, and pressure, respectively. We solved Equation (2) under the incompressibility condition ∇⋅v=0. The time evolution of the fluid is calculated using a Euler integration with a staggered grid; according to the velocity of the fluid, we can also move the cells by the equation of motion $r\left(t+\Delta t\right)=r\left(t\right)+V_{i}\left(t\right)\Delta t$, where $V_{i}\left(t\right)=\frac{\int v\left(r,t\right)\phi_{i}\left(r\right)\text{d}r}{\int \phi_{i}\left(r\right)\text{d}r}$ is the integrated velocity of the cell *i*. In Equation (2), the tensor *f^B^* indicates the random stress noise, which satisfies the fluctuation–dissipation theorem [26,34].

(3) $$\langle {f^{B}}_{i}\left(r,\;t\right)\;{f^{B}}_{j}\left(r^{\prime },\;t^{\prime }\right)\rangle =-2\eta k_{B}T\delta_{ij}\nabla^{2}\delta \left(r-r^{\prime }\right)\delta \left(t-t^{\prime }\right)$$

The stress term consists of two types of interactions. The first is the interaction derived from the interparticle potential. In this simulation, we assume that the short-range repulsion, which acts as the soft surface of the cell and prevents the penetration of cells into other nearby cells. In addition, we also assume that the attractive forces can be explained by van der Waals forces. For macroscopic objects like cells, it is necessary to integrate over all paired van der Waals interactions. In the case of monodisperse spherical bodies, Hamaker assumed the pair additivity of interaction potentials and derived the integrated van der Waals potential as follows:

(4) $$U_{\text{vdW}}\left(r\right)=-\frac{A_{\text{H}}}{\;12\;}\;\left[\frac{\sigma^{2}}{r^{2}-\sigma^{2}}+{\left(\frac{\;\sigma \;}{\;r\;}\right)}^{2}+\text{ln}\left(1-{\left(\frac{\;\sigma \;}{\;r\;}\right)}^{2}\right)\right]$$

This equation shows that the interaction range becomes longer in comparison with the case of unintegrated interaction of atoms (~1/*r*^6^). As a result, the attractive force acting on cells can be written as:

(5) $$F_{\text{vdW}}\left(r\right)=-U_{\text{vdW}}^{\prime }\left(r\right)=-\frac{A_{\text{H}}}{\;6\;\sigma \;}\;{\left(\frac{\;\sigma \;}{\;r\;}\right)}^{7}\;\left[1\;/{\left(1-{\left(\frac{\;\sigma \;}{\;r\;}\right)}^{2}\right)}^{2}\right]\;\frac{r}{\;r\;}$$

where σ denotes the interaction range, and *A*_H_ is the Hamaker constant. We set this quantity as the typical value *A*_H_/6*k_B_T* = 4. Since this potential diverges at the surface of cells, we introduced the short-range repulsive potential, which acts as the soft core of the cells and can be easily connected to the van der Waals potential shown in Equation (4). Therefore, we chose the Lennard–Jones-like potential *U_nm_*(*r*), whose derivative is given as $F_{nm}\left(r\right)=-U_{nm}^{\prime }\left(r\right)=\varepsilon \left[n{\left(\sigma /\;r\right)}^{n}-m{\left(\sigma /\;r\right)}^{m}\right]/\;r\;$, where the first term in parentheses indicates the short-range repulsion, and the second term indicates the short-range attraction to connect the attractive force shown in Equation (5). In the simulation, we set *n* = 36, *m* = 6.

Then, in order to connect the force at the cutoff radius *R*_cut_, the interparticle force was chosen as follows:

(6) $$F_{\text{tot}}\left(r\right)=\left\{ \begin{array}{cc} F_{nm}\left(r\right)-F_{nm}\left(R_{\text{cut}}\right)+F_{\text{vdW}}\left(R_{\text{cut}}\right) & \left(r\leq R_{\text{cut}}\right)\\ F_{\text{vdW}}\left(r\right) & \left(r>R_{\text{cut}}\right) \end{array}\right.$$

**Figure 6:**
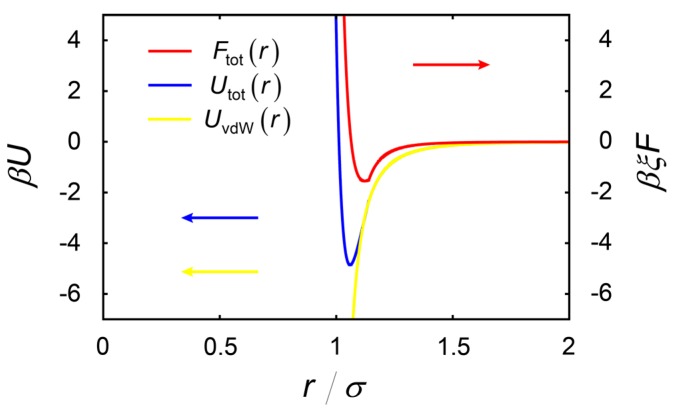
The interparticle potential (**blue line**) and the force (**red line**) acting on cells. There exists a soft repulsive force close to the cell wall. The **yellow line** is plotted according to Equation (4), and this potential could effectively describe the medium-ranged nature of the attractive potential.

In our simulation, we set *R*_cut_ = 1.14σ, and the force field is given as $f=-F_{\text{tot}}\;\phi /\int \phi \text{d}V$. The interparticle potential and force are plotted in Figure 6. From this plot, we can find that the interaction potential is connected smoothly at the cutoff radius and can describe both the medium-range attraction and the short-range repulsion.

The other interaction is the electrostatic force which is strongly affected by the local distribution of ions. In order to consider the local distribution of ions and its effect to the local stress we will introduce charges to this simulation method [35]. The concentration of ions of species α is given by *C*α and its valence is denoted as *Z*α. We also define the surface charges of cells as a continuous field quantity. When a cell *i* has surface charges *Q_i_*, the distribution of charges are considered to distribute uniformly and is written as $\rho_{i}\left(r\right)=Q_{i}{\left|\nabla \phi_{i}\left(r\right)\right|}^{2}/\;\int {\left|\nabla \phi_{i}\left(r\right)\right|}^{2}\text{d}r$; as *Q_i_*, we used the scaled value according to the experiments [27,28]. By using these quantities, the electrostatic force is expressed as $f_{el}=-\rho_{tot}\;\nabla \psi$ , where *ρ_tot_* indicates the total charge density, which is written as $\rho_{tot}=\sum_{i}\rho_{i}+\sum_{\alpha }ez_{\alpha }C_{\alpha }$. In this formula, the electrostatic potential *ψ* is calculated from the Poisson equation $\varepsilon_{r}\varepsilon_{0}\Delta \psi =-\rho_{tot}$ where *ε*_0_ and *ε*_r_ denote the permittivity of vacuum and relative dielectric constant, respectively. The time evolution of ions surrounding cells satisfies the following diffusion equation.

(7) $$\left(\frac{\partial \;}{\partial \;t}+v\cdot \nabla \right)C_{\alpha }=\nabla \cdot \left[LC_{\alpha }\nabla \left(k_{B}T\ln C_{\alpha }+ez_{\alpha }\psi +k_{B}T\chi \sum_{i}\phi_{i}\right)\right]+\theta \left(r,\;t\right)$$

In this equation, *k*_B_ is the Boltzmann constant, and *T* is the temperature. We set the temperature at room temperature *T* = 30 °C. The diffusion constant of ions *D*_ion_ satisfies *D*_ion_ = *k*_B_*TL*, where we chose *D*_ion_ as the typical value *D*_ion_ = 10^−9^ (m^2^/s), and *χ* is the artificially introduced parameter, which prevents ions from penetrating into the cells, and we set to *χ* = 3. In the final term of this equation, the thermal fluctuation is considered, satisfying the fluctuation–dissipation theorem as follows [34,36,37]:

(8) $$\langle \theta \left(r,\;t\right)\;\theta \left(r^{\prime },\;t^{\prime }\right)\rangle =-2\;L\;\nabla C_{\alpha }\nabla \delta \left(r-r^{\prime }\right)\delta \left(t-t^{\prime }\right)$$

All of these equations are scaled using the unit length *λ* and time *τ*. $\lambda =Ae^{2}/4\pi \varepsilon_{0}\varepsilon_{r}k_{B}T$ was taken as the unit length, and we could change the constant parameter A and calculate the appropriate system size. In this simulation, we took the interfacial thickness as the unit length *ξ* = *λ*. The unit time is also defined from the relaxation time for fluids as $\tau =\rho \lambda^{2}/\;\eta_{sol}=\lambda^{2}/\;\nu$, where ν is the kinematic viscosity coefficient of the solvent. We solved these equations with periodic boundary conditions.

### 3.2. Dynamics of a Single Cell

**Figure 7:**
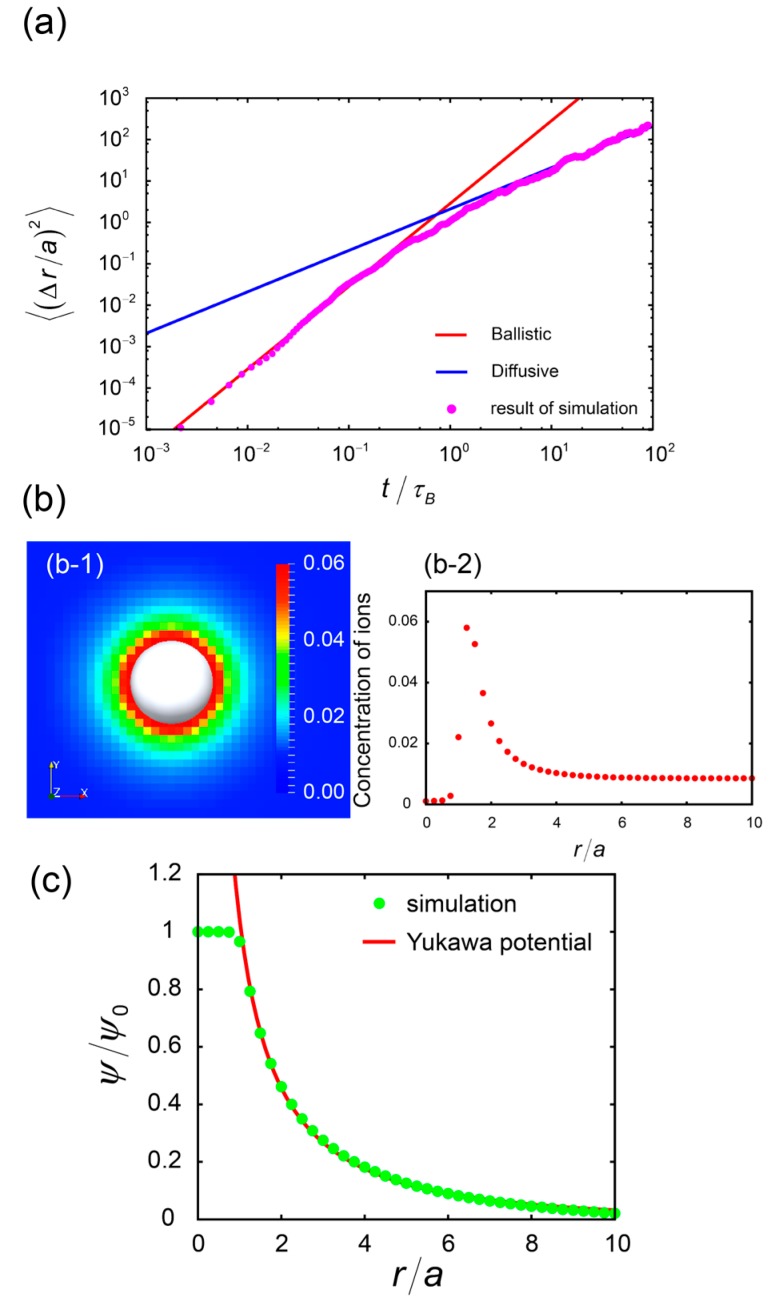
(**a**) Brownian motion of charged cells from the calculation of the mean square displacement of cells. The Brownian motion of cells exhibited a continuous change from ballistic (below *τ*_B_) to diffusive motion (above *τ*_B_). The horizontal axis indicates time scaled by the Brownian time, and the vertical axis indicates the MSD scaled by the radius of cells. The red and blue solid lines represent the ballistic motion and diffusional motion, which are characterized by $\left(3k_{B}T/m\right)t^{2}$ and 6Dt, respectively; (**b**) The concentration distribution of ions surrounding charged cells: (**b-1**) two-dimensional profile of counter ion concentration and (**b-2**) concentration profile of counter ions surrounding a cell. This shows the artificially introduced potential $k_{B}T\chi$ prevents ions from penetrating into the cell; (**c**) Electrostatic potential of charged cells. Screened-coulomb or Yukawa potential allowed us to fit the profile. All of the results in this figure were obtained from three-dimensional simulations with box sizes of 128 × 128 × 128.

In order to confirm the dynamic properties, Figure 7a plots the temporal change in the mean square displacement (MSD) of the cells. The simulation of each run was performed in three dimensions with a sufficiently large simulation box (128 × 128 × 128). In Figure 7a, the average of the results of 16 distinct simulation runs is shown. In this figure the vertical and horizontal axes were scaled by radius of cells a and the Brownian time $\tau_{B}=a^{2}/\;D_{cell}$, respectively. We assumed that cells had a spherical shape, and the diffusion constant of the cells was estimated from the Einstein relation $D_{cell}=\frac{k_{B}T}{6\pi \eta a}$. Below *τ*_B_, the MSD is proportional to *t*^2^, since the cell is kicked by the surrounding fluid molecules, which move by the fluctuation, and cells move with a constant velocity $v=\sqrt{{3k_{B}T/\;m}_{C}}$ for this short-time regime, where *m*_c_ denotes the mass of the cell in this regime. This stage is called as ballistic regime. On the other hand, the diffusional process dominates above *τ*_B_ (at long times), and the MSD grows in proportion to the time $\langle {\left(\Delta r\right)}^{2}\rangle =6D_{cell}t$. This crossover is well-described at *t* = *τ*_B_.

### 3.3. Distribution of Ions and Electrostatic Behavior

Electrostatic behaviors of ions and cells are also confirmed. Figure 7b,c show the distribution of ions surrounding the cell and the behavior of electrostatic potential from the center of the cell. The conditions for simulation were the same as in the previous section, and we only considered the effects of counter ions of the cell. Ions are strongly bound to the surface of the cell, and the concentration of ions gradually decreases as the distance from the cell becomes greater. Figure 7b-2 clearly shows that the artificially introduced potential prevents ions from penetrating into the cell. The bounded ions screen the electrostatic potential around the cell, which is known to be described by the following screened Coulomb (or Yukawa) potential.

(9) $$\beta \psi \left(r\right)=\beta \varepsilon_{\text{Yuk}}\frac{\text{exp}\left(-\kappa \left(r/\sigma -1\right)\right)}{r/\sigma }$$

where $\beta \varepsilon_{\text{Yuk}}$ is the magnitude of the Yukawa repulsion in the units $\beta =1/k_{B}T$, and *κ* is the inverse Debye screening length in units of the hard-sphere diameter *σ* = 2*a*. Figure 7c shows that the simulation results of single particles (green dots) were well described by this Yukawa potential (red solid line).

### 3.4. Langevin Dynamics Simulation

The Langevin dynamics (LD) simulation is based on the Langevin equation of motion:

(10) $$m\frac{dv}{dt}=F-F_{\text{drag}}+F_{\text{B}}$$

LD omits the solvent degrees of freedom. However, the Stokes’ drag force $F_{\text{drag}}=-\zeta v$, which mimics the solvent surrounding cells, is introduced in order to account for the viscous aspects of solvents. Here, we assumed cells had a spherical shape, and ζ is written as ζ=6πηa. The notation and value of each variable and the interparticle force $F=-\nabla U_{\text{tot}}$ used here are the same as those described in Section 3.1.

## 4. Conclusions

We focused on the initial stage of network formation of vascular endothelial cells, which is not affected by chemoattractants, such as VEGF. From simple observations, we found that cells formed chain-like structures as a primary phenomenon of network formation. In order to elucidate the mechanisms mediating this process, we performed a hybrid simulation of the mesh-less dynamics of cells and a continuous field method. The continuous model allowed us to model the other degrees of freedom, such as hydrodynamic interactions and the distribution of ions.

From these simulations, we found that hydrodynamic interactions slowed the compaction of clusters and that the lifetime of the chains became longer. This structure was stabilized by the effects of charges. The Coulomb repulsion of the effective interparticle potential between cells prevented chains from folding, and ions that bound to the cells further stabilized the chains. Therefore, the initial configuration of the network should not be treated as homogeneous, but should instead be considered as a directional precursor. Our findings suggested that chemoattractants, such as VEGF, may be necessary for the later stages of network formation, as has been shown in multiple studies [1,2,38]; however, the initial configuration of cells will also affect the speed at which the networks form and the final morphologies of the networks.

Our simulation also suggested that it is important to add salts to screen long-range interactions for generating precursors of networks of vascular endothelial cells. This may affect the threshold of the percolation transition [8]. Thus, these purely physical effects like electrostatic forces, hydrodynamic interactions and elastic forces should be considered in other biological structural transformations, such as cell division, tube formation of endothelial cells, and late-stage network formation. Actually, in the late stages of network formation, the other factors for example the size distribution of cells and the elasticity of cells should also be considered. This will be future work.

Finally, we expect that the simulation method presented above will be applicable to other physical and biological processes, like charged colloidal systems, and the later stages of network formation, during which VEGF or other chemicals are present and play a role in forming networks. Additional experiments and simulations are necessary to elucidate the many features of these morphogenic processes.

## Supplementary Materials

Supplementary materials can be found at http://www.mdpi.com/1422-0067/16/12/26149/s1.
