# Supplementary material for: Primary Phenomenon in the Network Formation of Endothelial Cells: Effect of Charge
Source: Int J Mol Sci. 2015 Dec 7;16(12):29148–60. doi: 10.3390/ijms161226149 (PMC4691096; doi:10.3390/ijms161226149)
Supplement: Supplementary file 1 [file ijms-16-26149-s001.pdf]

Supplementary Materials: Primary Phenomenon in the Network Formation of Endothelial Cells: Effect of Charge

Shunto Arai

Observation of Endothelial Cells

In Figure 1, we made simple observations of the suspensions using a phase-contrast microscope. For this observation, we prepared cells and substrates according to the following steps (for more details, see Matsunaga *et al.* [14]).

1. Matrigel was stored at 4 °C and was melted before the experiments. The Matrigel was generally stored in a refrigerator and kept at −80° C. The dishes were also cooled to the same temperature.
2. Matrigel (120 μL) was added to the dish and heated at 37 °C for 10 min to form a gel. The gels were swollen by adding 1 mL of a solution containing epidermal growth factor (EGF)-2.
3. Human umbilical vein endothelial cells (HUVECs) were detached using trypsin and incubated for 10 min with endothelial cell basal medium (EBM™)-2, which did not contain VEGF.
4. After mixing endothelial cell growth medium (EGM™)-2 with the prepared cells, the cells were centrifuged and resuspended.
5. Cells were then seeded on the substrate prepared in step 2. Medium was added to reach a final volume of 2 mL, and suspensions were observed.
